# Global Transcriptome Analysis of *Aedes aegypti* Mosquitoes in Response to Zika Virus Infection

**DOI:** 10.1128/mSphere.00456-17

**Published:** 2017-11-22

**Authors:** Kayvan Etebari, Shivanand Hegde, Miguel A. Saldaña, Steven G. Widen, Thomas G. Wood, Sassan Asgari, Grant L. Hughes

**Affiliations:** a Australian Infectious Disease Research Centre, School of Biological Sciences, The University of Queensland, Brisbane, Queensland, Australia; b Department of Pathology, University of Texas Medical Branch, Galveston, Texas, USA; c Department of Microbiology and Immunology, University of Texas Medical Branch, Galveston, Texas, USA; d Department of Biochemistry and Molecular Biology, University of Texas Medical Branch, Galveston, Texas, USA; e Department of Pathology, Institute for Human Infections and Immunity, Center for Tropical Diseases, Center for Biodefense and Emerging Infectious Disease, University of Texas Medical Branch, Galveston, Texas, USA; Icahn School of Medicine at Mount Sinai

**Keywords:** *Aedes aegypti*, RNA-Seq, Zika virus, behavior, long noncoding RNA, microRNA, odorant binding protein, transcriptome

## Abstract

Zika virus (ZIKV) of the *Flaviviridae* family is a recently emerged mosquito-borne virus that has been implicated in the surge of the number of microcephaly instances in South America. The recent rapid spread of the virus led to its declaration as a global health emergency by the World Health Organization. The virus is transmitted mainly by the mosquito *Aedes aegypti*, which is also the vector of dengue virus; however, little is known about the interactions of the virus with the mosquito vector. In this study, we investigated the transcriptome profiles of whole *A. aegypti* mosquitoes in response to ZIKV infection at 2, 7, and 14 days postinfection using transcriptome sequencing. Results showed changes in the abundance of a large number of transcripts at each time point following infection, with 18 transcripts commonly changed among the three time points. Gene ontology analysis revealed that most of the altered genes are involved in metabolic processes, cellular processes, and proteolysis. In addition, 486 long intergenic noncoding RNAs that were altered upon ZIKV infection were identified. Further, we found changes of a number of potential mRNA target genes correlating with those of altered host microRNAs. The outcomes provide a basic understanding of *A. aegypti* responses to ZIKV and help to determine host factors involved in replication or mosquito host antiviral response against the virus.

**IMPORTANCE** Vector-borne viruses pose great risks to human health. Zika virus has recently emerged as a global threat, rapidly expanding its distribution. Understanding the interactions of the virus with mosquito vectors at the molecular level is vital for devising new approaches in inhibiting virus transmission. In this study, we embarked on analyzing the transcriptional response of *Aedes aegypti* mosquitoes to Zika virus infection. Results showed large changes in both coding and long noncoding RNAs. Analysis of these genes showed similarities with other flaviviruses, including dengue virus, which is transmitted by the same mosquito vector. The outcomes provide a global picture of changes in the mosquito vector in response to Zika virus infection.

## Abstract (precis)

Vector-borne viruses pose great risks to human health. Zika virus has recently emerged as a global threat, rapidly expanding its distribution. Understanding the interactions of the virus with mosquito vectors at the molecular level is vital for devising new approaches in inhibiting virus transmission. In this study, we embarked on analyzing the transcriptional response of *Aedes aegypti* mosquitoes to Zika virus infection. Results showed large changes in both coding and long noncoding RNAs. Analysis of these genes showed similarities with other flaviviruses, including dengue virus, which is transmitted by the same mosquito vector. The outcomes provide a global picture of changes in the mosquito vector in response to Zika virus infection.

## INTRODUCTION

Flaviviruses are a group of arthropod-borne viruses (arboviruses) that impose huge burdens on global animal and human health. The best-known examples of flaviviruses that cause diseases in humans are yellow fever, West Nile, dengue, and Zika viruses. Zika virus (ZIKV) has been the most recent mosquito-borne virus to emerge. While it was first reported in 1952 from Uganda (1), the virus has spread rapidly across the Pacific and the Americas in the last 10 years with recent outbreaks in South America (2). The clinical symptoms are variable, ranging from no or mild symptoms to severe neurological disorders such as microcephaly in infants born from infected mothers or Guillain-Barré syndrome in adults (reviewed in references 2 and 3). The virus is mainly transmitted among humans by the bites of mosquito species of the genus *Aedes*, in particular *Aedes aegypti*, when it takes a blood meal from infected individuals. The virus first infects the midgut cells of the mosquito and then disseminates into other tissues, finally reaching the salivary glands, where it continues to replicate and is eventually transmitted to other human hosts upon subsequent blood feeding events (4).

It is thought that infection by flaviviruses does not cause any detrimental pathological effects on the mosquito vectors (5), reflecting evolutionary adaptations of the viruses with mosquitoes through intricate interactions, which involve optimal utilization of host factors for replication and avoidance of overt antiviral responses. However, a number of studies have shown major transcriptomic changes in the mosquito vectors in response to flavivirus infection. These changes suggest regulation of a wide range of host genes involved in classical immune pathways, RNA interference (RNAi), metabolism, energy production, and transport (6–13). In addition, mosquito small and long noncoding RNAs have also been shown to change upon flavivirus infection (14, 15).

Recently, we showed that the microRNA (miRNA) profile of *A. aegypti* mosquitoes is altered upon ZIKV infection at different time points following infection (16). Here, we describe the transcriptional response of *A. aegypti* whole mosquitoes to ZIKV infection at the same time points postinfection. Consistent with previous studies of other arboviruses, we found that the abundance of a large number of genes was altered following ZIKV infection.

## RESULTS AND DISCUSSION

### *A. aegypti* transcriptome RNA sequencing (RNA-Seq) data analysis.

RNA-Seq using Illumina sequencing technology was performed on poly(A)-enriched RNAs extracted from ZIKV-infected and noninfected *A. aegypti* mosquitoes at 2, 7, and 14 days postinfection (dpi). Total numbers of clean paired reads varied between 43,486,502 and 60,486,566 reads per library among the 18 sequenced RNA samples. More than 96% of reads mapped to the host genome with around 80% of counted fragments mapped to gene regions and 20% to intergenic areas of the genome (see Table S1 in the supplemental material).

10.1128/mSphere.00456-17.2 TABLE S1  RNA read summary in ZIKV-infected and noninfected libraries. Download TABLE S1, DOCX file, 0.1 MB. Copyright © 2017 Etebari et al. 2017 Etebari et al. This content is distributed under the terms of the Creative Commons Attribution 4.0 International license.

**FIG 1:**
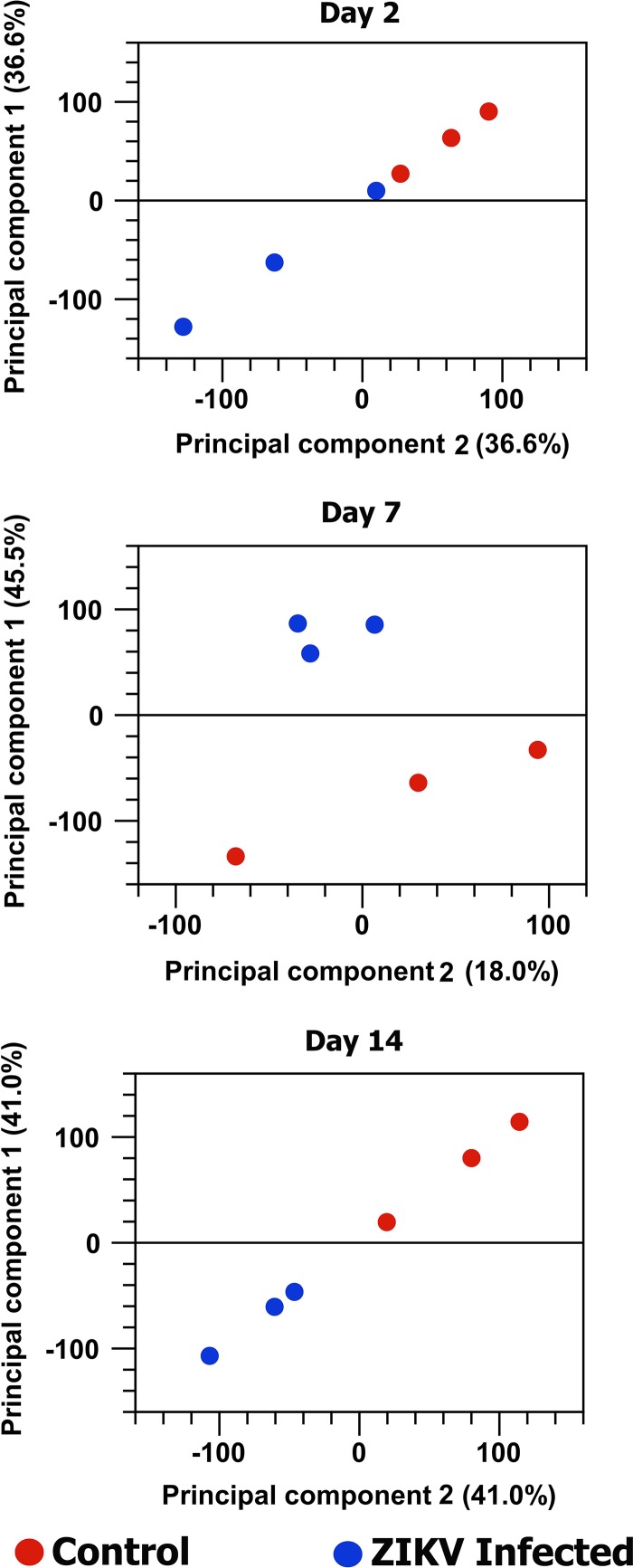
The principal-component analysis of the effect of ZIKV infection on *A. aegypti* transcriptome at three different time points postinfection. The normalized log count per million (cpm) was used as an expression value in this analysis.

Principal-component analysis (PCA) of the RNA-Seq data at each time point distributed all biological replicates of ZIKV-infected and noninfected samples in two distinct groups, although the differences were more subtle at 2 days postinfection, in which one of the ZIKV-infected biological replicates was relatively close to the control group (Fig. 1).

**FIG 2:**
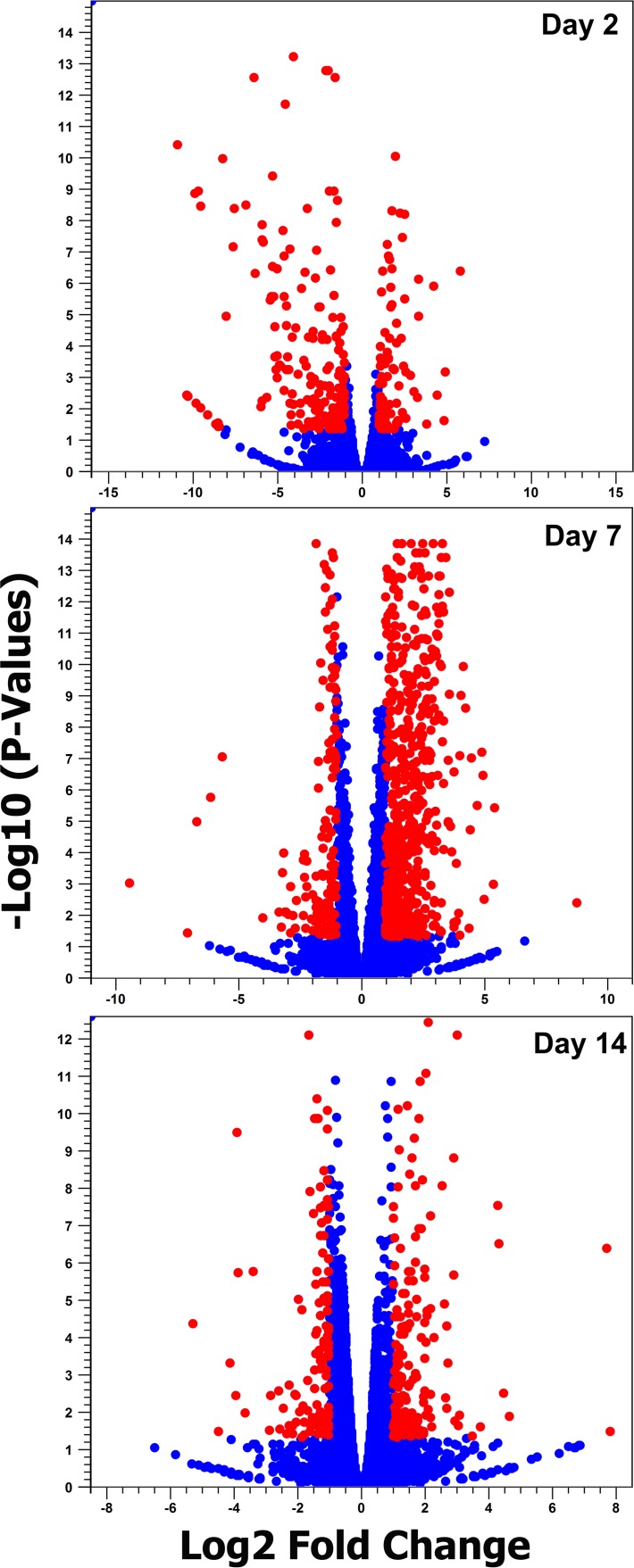
Volcano plot analysis. Red circles indicate mRNAs differentially expressed in response to ZIKV infection (fold change of >2 and FDR of <0.05).

**FIG 3:**
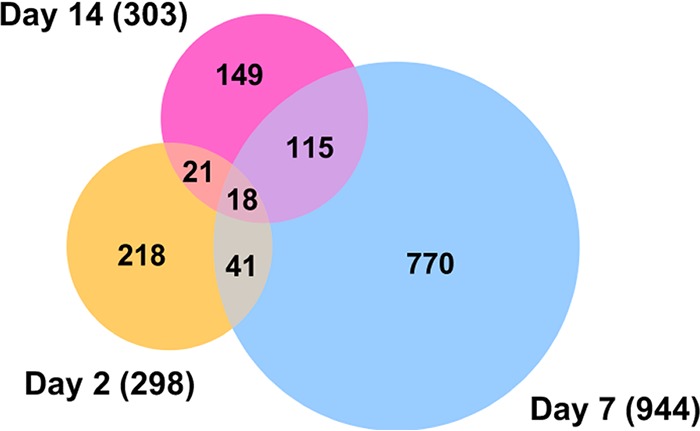
Venn diagram representing the number of differentially expressed coding genes at three different time points post-ZIKV infection. Profound alteration in gene expression was observed at 7 dpi, and more common differentially expressed genes were found between day 7 and day 14 samples.

Analysis and comparison of mRNA expression profiles of *A. aegypti* mosquitoes at different time points following ZIKV infection revealed that in total 1,332 genes had changes of 2-fold or more in either direction (Fig. 2; details in Table S2). Among the three time points, the highest number of changes occurred at 7 dpi with 944 genes showing alteration in their transcript levels. The numbers of genes altered at 2 and 14 dpi were very close, 298 and 303, respectively (Fig. 3). These trends were anticipated, as we expected to see lower gene expression alteration at 2 dpi and 14 dpi due to the low level of infection in the mosquito body at 2 dpi and advanced stages of virus replication at 14 dpi, while at 7 dpi the virus is still at its proliferative stage, infecting various tissues of the mosquito. In a previous study that explored the effect of dengue virus type 2 (DENV-2) on the *A. aegypti* transcriptome using RNA-Seq (11), the number of genes altered was the highest at 4 dpi (151, combining carcass and midgut), compared to 1 dpi, which showed the lowest number of changes (40 genes) followed by 14 dpi (82 genes).

10.1128/mSphere.00456-17.3 TABLE S2  Differentially expressed transcripts in response to ZIKV at 2, 7, and 14 dpi. Download TABLE S2, XLSX file, 0.2 MB. Copyright © 2017 Etebari et al. 2017 Etebari et al. This content is distributed under the terms of the Creative Commons Attribution 4.0 International license.

**FIG 4:**
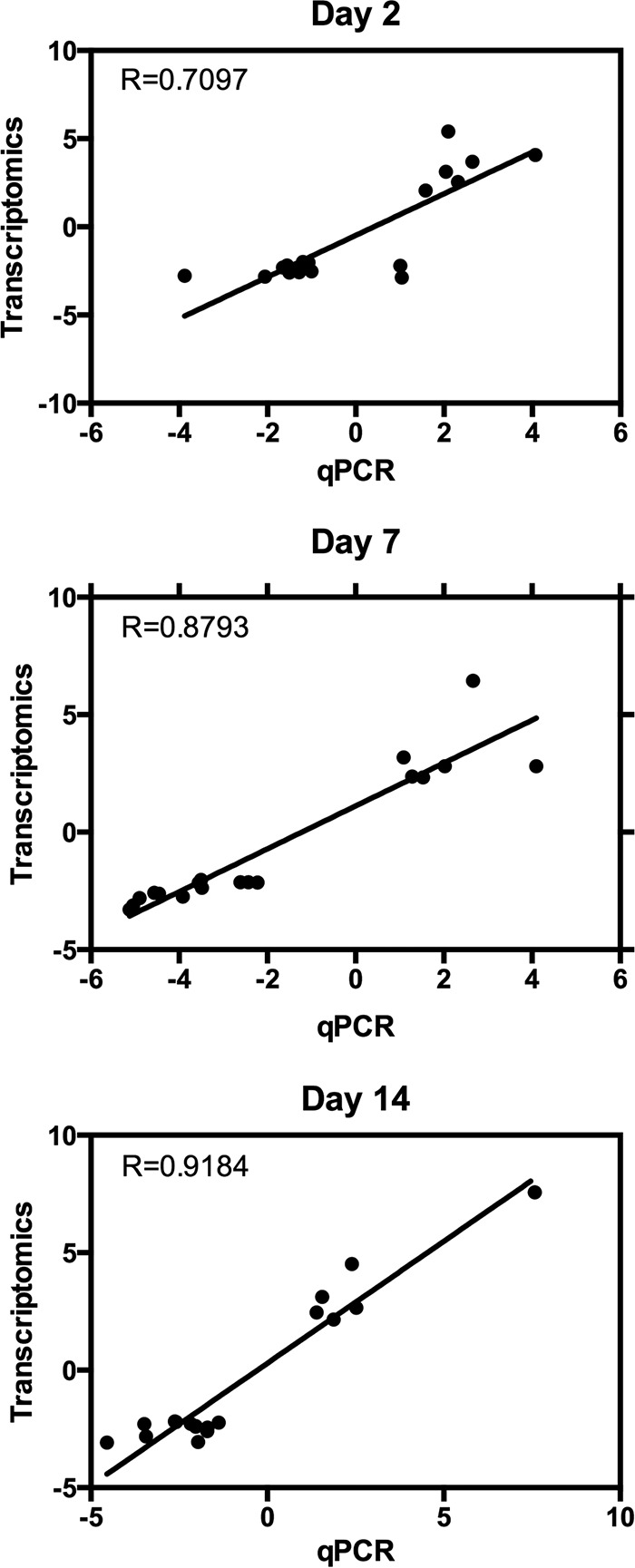
Validation of RNA-Seq data analysis by RT-qPCR. The 18 genes that were differentially expressed at all time points were validated by RT-qPCR at 2, 7, and 14 days postinfection. Overall, all time points showed consistency between the two methods in their trends of depletion or enrichment.

Comparison of the transcriptome profiles showed 18 overlapping genes among the three time points (Fig. 3; listed in Table 1). Twelve of these common genes were depleted, and only six were enriched, which were angiotensin-converting enzyme (AAEL009310), serine-type endopeptidase (AAEL001693), phosphoglycerate dehydrogenase (AAEL005336), cysteine dioxygenase (AAEL007416), and two hypothetical proteins. To validate the analysis of the RNA-Seq data, we used reverse transcription-quantitative PCR (RT-qPCR) analysis of the 18 genes. Overall, all expression values showed consistency between the two methods and had a positive linear correlation (Pearson correlation; day 2, *R*^2^ = 0.7097, *P* < 0.0001; day 7, *R*^2^ = 0.8793, *P* < 0.0001; day 14, *R*^2^ = 0.9184, *P* < 0.0001) (Fig. 4).

**TABLE 1:** List of *A. aegypti* differentially expressed genes common to all three time points post-ZIKV infection

Gene identifier	Gene description	Day 2		Day 7		Day 14	
		Fold change	FDR *P* value	Fold change	FDR *P* value	Fold change	FDR *P* value
AAEL009310	Angiotensin-converting enzyme	5.41	3.66E−8	3.18	4.61E−4	2.65	4.81E−3
AAEL001693	Serine-type endopeptidase	4.08	8.02E−5	2.33	4.74E−3	2.46	3.81E−3
AAEL005336	d-3-Phosphoglycerate dehydrogenase	2.06	0.02	2.81	3.00E−9	2.15	1.66E−3
AAEL010153	Protein bicaudal C	−2.59	9.34E−3	−2.81	0	−3.08	8.26E−13
AAEL003688	Conserved hypothetical protein	−2.21	3.40E−3	−2.13	5.98E−12	−2.23	3.00E−8
AAEL005501	B-box-type zinc finger protein nCL-1	−2.87	4.86E−5	−2.13	5.55E−10	−2.6	1.72E−6
AAEL017329	B-box-type zinc finger protein nCL-1	−2.52	6.34E−4	−2.14	8.32E−8	−2.44	9.77E−9
AAEL005850	Hormone receptor-like in 4 (nuclear receptor)	−2.57	8.90E−4	−2.75	3.66E−13	−3.06	1.23E−8
AAEL007416	Cysteine dioxygenase	3.12	7.28E−3	2.37	0.02	4.52	5.85E−8
AAEL010086	DNA replication licensing factor MCM4	−2.2	5.94E−3	−2.15	2.33E−10	−2.4	1.90E−7
AAEL010228	Conserved hypothetical protein	2.54	0.03	6.45	1.29E−9	3.12	3.10E−6
AAEL010644	Ribonucleoside-diphosphate reductase large chain	−2.3	0.03	−2.62	0	−2.28	5.52E−7
AAEL011811	DNA replication licensing factor MCM3	−2.03	8.63E−3	−2.37	0	−2.21	1.90E−7
AAEL012339	Cdk1	−2	4.40E−3	−2.58	1.05E−7	−2.82	5.07E−8
AAEL013338	Lethal (2) essential for life protein, l2efl	−2.78	5.28E−5	−3.3	0	−2.38	8.43E−8
AAEL013577	Conserved hypothetical protein	3.7	5.81E−4	2.81	0.02	7.57	2.13E−6
AAEL013602	Laminin gamma-3 chain	−2.31	6.85E−3	−2.03	2.80E−3	−2.29	4.66E−4
AAEL003797	Hypothetical protein	−2.82	4.43E−3	−3.12	9.61E−11	−2.18	2.47E−3

### Differentially abundant transcripts and comparisons with other flaviviruses.

When concentrating on genes with 10-fold differential expression and statistical significance relative to control mosquitoes, 101, 54, and 17 genes showed changes at 2, 7, and 14 dpi, respectively (Table S2, dark blue font). After removal of hypothetical proteins, those with known functions are listed in Table 2. Interestingly, while the total number of genes showing differential abundance was higher at 7 dpi (Fig. 3), more genes showed 10-fold or greater changes at 2 dpi than at 7 dpi (101 versus 54, respectively).

**TABLE 2:** List of *A. aegypti* differentially expressed genes with more than 10-fold change specific to each time point post-ZIKV infection

Name	Gene identifier	Description	Fold change^a^		
			Day 2	Day 7	Day 14
AAEL011539	5574950	Metalloproteinase, putative	**56.2**	−1.1	3.31
AAEL013298	5577578	Serine protease, putative	**22.08**	1.86	4.74
AAEL007601	5569396	Trypsin 5G1-like	**18.98**	3.46	3.06
AAEL013707	5578506	Trypsin 5G1-like	**10.07**	6.24	1.81
AAEL011260	5574623	Protein D3	**−10.95**	3.1	−0.18
AAEL011954	5575620	Elongation of very-long-chain fatty acids protein 7	**−11.96**	1.56	1.14
AAEL014312	5564093	Cubilin homolog	**−12.1**	8.42	1.87
AAEL010965	5574152	Cubilin homolog	**−12.49**	1.4	−1.61
AAEL010139	5572918	Putative defense protein 1	**−14.85**	34.42	0.9
AAEL003094	5577074	Glycoprotein, putative	**−16.59**	6.43	−1.29
AAEL011491	5574891	General odorant binding protein 67	**−17.05**	−2.93	0.46
AAEL001487	5570904	General odorant binding protein 45-like	**−17.49**		2.66
AAEL004947	5565723	Elongation of very-long-chain fatty acids protein 4	**−18.74**	−2.25	5.11E−3
AAEL005090	5565985	Cysteine-rich venom protein, putative	**−18.75**	3.74	
AAEL010875	5574034	General odorant binding protein 45-like	**−20.22**		
AAEL007096	5568731	Major royal jelly protein 3	**−21.97**	1.01	0.67
AAEL010848	5574004	Major royal jelly protein 5	**−23.73**	1.45	−0.67
AAEL010872	5574030	General odorant binding protein 45-like	**−27.81**	−8.89	0.38
AAEL011808	5575404	Glucose dehydrogenase (flavin adenine dinucleotide, quinone)	**−29.51**	−1.01	3.95E−3
AAEL006398	5567938	OBP32, odorant binding protein OBP32	**−31.43**	1.71	
AAEL006393	5567943	OBP28, odorant binding protein OBP28	**−35.93**	−6.24	
AAEL005925	5567269	Geranylgeranyl pyrophosphate synthase	**−38.51**	3.74	2.96E−3
AAEL006396	5567937	OBP31, odorant binding protein OBP31	**−56.46**	−3.6	−1.31
AAEL003511	5578352	General odorant binding protein 45-like	**−59.39**	−1.75	0.79
AAEL015262	5566792	Phosphatidylethanolamine-binding protein, putative	**−59.59**	1.4	0.56
AAEL000796	5566894	General odorant binding protein 45-like	**−302.47**	3.74	2.66
AAEL015052	5566038	Steroid receptor RNA activator 1	**−358.45**	3.11	7.73
AAEL000827	5566899	General odorant binding protein 45-like	**−362.89**	−1.75	
AAEL000846	5566895	General odorant binding protein 45-like	**−397.26**	2.42	0.51
AAEL000833	5566896	General odorant binding protein 45-like	**−739.97**	2.42	1.27
AAEL000835	5566905	General odorant binding protein 45-like	**−811.93**		−0.78
AAEL000837	5566897	General odorant binding protein 45-like	**−883.73**	−1.01	2.67
AAEL000701	5565919	39S ribosomal protein L4, mitochondrial	1.25	**438.21**	
AAEL015019	5565969	Protein artichoke	−1.43	**42.54**	1.31
DEFD	5579095	Defensin A-like	−8.11	**31.28**	−4.25
AAEL014386	5564283	Serine protease Easter	−2.06	**30.31**	2.23
DEFA_AEDAE	5579099	Defensin A	−7.35	**21.99**	−4.45
AAEL015430	5579444	Serine protease, putative	−1.19	**21.79**	−1.05
AAEL015639	5579270	Transferrin	−3.55	**19.09**	−1.67
AAEL014005	5579131	Clip-domain serine protease, putative	−2.03	**17.69**	1.47
CTLMA15	5563672	C-type lectin, 37 Da	−1.1	**16.19**	3.91
TRY5_AEDAE	5578510	Trypsin 5G1	−1.15	**15.52**	2.13
DEFC_AEDAE	5579094	Defensin C	−8.23	**14.5**	−5.59
AAEL013640	5578322	Lung carbonyl reductase	3.51	**13.7**	1.49
AAEL010429	5573346	Protein G12	5.92	**13.51**	25.07
AAEL002726	5575756	37-kDa salivary gland allergen Aed a 2-like	1.09	**12.19**	1.63
AAEL015458	5579417	Transferrin	−9.02	**11.93**	−1.19
AAEL013542	5578161	Elongation of very-long-chain fatty acids	−1.65	**11.85**	2.91
AAEL002672	5575549	Matrix metalloproteinase-19	−1.25	**11.49**	1.36
AAEL013990	5579047	Hexamerin-1.1	−1.16	**10.97**	1.96
AAEL005787	5567041	Serine protease Easter	−1.05	**10.3**	1.72
AAEL015628	5579281	Glycine dehydrogenase	2.92	**10.07**	1.84
AAEL004134	5564162	Lupus la ribonucleoprotein	2.24	**−69.95**	−2.86
AAEL003946	5563782	28S ribosomal protein S33, mitochondrial	−2.19	**−101.56**	1.57
AAEL009497	5572080	Probable phosphomannomutase	−1.25	**−676.56**	−3.51
AAEL015052	5566038	Steroid receptor RNA activator 1	−358.45	3.11	**212.47**
AAEL010429	5573346	Protein G12	5.92	13.51	**25.07**
AAEL009435	5571953	Adhesion-regulating molecule 1	−1.34	−1.21	**13.51**
AAEL002613	5575308	Peritrophin-48	3.23	−3.98	**11.35**
ATT	5578028	Attacin-B	4.4	−1.42	**−10.49**
AAEL007040	5568687	Protein lozenge, transcript variant X3	−1.83	45.7	**−12.57**
AAEL011550	5574942	Seminal metalloprotease 1	−1.93	1.71	**−22.18**
CUSOD3_a	5573744	Superoxide dismutase (Cu-Zn)	1.03	1.18	**−39.11**

a Values in bold are fold changes greater than 10 specific to each time point.

At 2 dpi, transcripts of eight genes were enriched with a metalloproteinase (AAEL011539) showing a 56-fold increase in abundance, a serine protease (AAEL013298) increasing 22-fold, and two trypsins (AAEL007601 and AAEL013707) with 19- and 10-fold increases, respectively. We also saw that two phosphatidylethanolamine-binding proteins, two cubulin proteins, and a cysteine-rich venom protein were altered at this time point. However, most strikingly, we observed suppression of 14 odorant binding proteins at 2 dpi, with several of these transcripts being massively reduced (around 800-fold) (Table 2). Furthermore, other odorant binding protein transcripts were enhanced (by 2-fold or greater) at 7 and 14 dpi (Fig. S1), indicating that ZIKV may have the capacity to alter the behavior of the mosquito, potentially suppressing host-seeking in early stages of the infection and encouraging host-seeking when the mosquito is infectious. Dengue virus is known to alter host-seeking behaviors and feeding efficiency (17, 18), and microarray analysis of mosquitoes with salivary gland infections found several odorant binding protein transcripts that were enriched in this late stage of infection (14 dpi) (19). Similarly, there is evidence that malaria parasites suppress the host-seeking tendencies of the mosquito early in infection but encourage host-seeking at later stages when the mosquito can transmit the parasite (20–22). The transcription patterns that we observed here with ZIKV are consistent with these observations from dengue and malaria infection of mosquitoes, but further behavioral studies are required to confirm this intriguing finding.

10.1128/mSphere.00456-17.1 FIG S1  Expression levels of odorant binding protein transcripts at days 2, 7, and 14. Fold change values are given in log2 scale. Download FIG S1, PDF file, 0.3 MB. Copyright © 2017 Etebari et al. 2017 Etebari et al. This content is distributed under the terms of the Creative Commons Attribution 4.0 International license.

At 7 dpi, 34 genes showed enrichment of 10-fold or more, including clip-domain serine proteases, defensins, transferrins, hexamerin, C-type lectin, and serine proteases, which are implicated in immune responses. At this time point, only seven genes were depleted. The number of genes that were differentially expressed by 10-fold or more at 14 dpi was small, with eight genes showing enrichment and eight genes showing depletion. The highest enrichment (212-fold) was steroid receptor RNA activator 1 (AAEL015052), while peritrophin, attacin, and superoxide dismutase were among the depleted genes (Table 2).

Previous studies have shown alteration of mRNA transcript levels in *A. aegypti* mosquitoes infected with DENV and a couple of other flaviviruses. Using microarray analysis, Colpitts et al. found that 76 genes showed 5-fold or greater changes in DENV-infected mosquitoes over 1, 2, and 7 dpi (13). Their study, which also included responses of *A. aegypti* to West Nile virus (WNV) and yellow fever virus (YFV), found that commonly 20 and 15 genes were differentially enriched and depleted, respectively, between the three flaviviruses at day 1 postinfection. Considering utilization of two different techniques in the work of Colpitts et al. (microarray) and in this study (RNA-Seq) and differences between the time points chosen, proper comparison of changes in transcript levels and fold changes cannot be done. However, when we mapped all the differentially expressed genes (2-fold or more) from the work of Colpitts et al. against our data (Table S2), we found that 364 genes from our study showed differential expression at least at one time point that overlaps the other three viral infections (Table S3).

10.1128/mSphere.00456-17.4 TABLE S3  Comparison of transcripts modulated by ZIKV to those modulated by dengue virus, West Nile virus, and yellow fever virus identified by Colpitts et al. (13). Download TABLE S3, XLSX file, 0.1 MB. Copyright © 2017 Etebari et al. 2017 Etebari et al. This content is distributed under the terms of the Creative Commons Attribution 4.0 International license.

In a follow-up study using the data from the above study (1, 2, and 7 dpi) (13), Londono-Renteria et al. identified 20 top differentially regulated transcripts in YFV-, DENV-, and WNV-infected *A. aegypti* mosquitoes (23). Out of these 20 genes, five of them were also found to be changed in ZIKV-infected mosquitoes in our study. These were the cysteine-rich venom proteins (AAEL005098, AAEL005090, AAEL000379, and AAEL000356) by about 9-, 18-, 25-, and 150-fold depletion at 2 dpi, respectively, and an unknown protein (AAEL013122) by 390-fold depletion at 2 dpi. While pairwise comparison is not really possible between the two studies, comparing data from 2 dpi showed that AAEL005090 (in the case of DENV), AAEL005098 and AAEL000356 (in the case of YFV and WNV, respectively), and AAEL013122 (in the case of DENV) changed in the same direction as in ZIKV infection. Another study also found a number of cysteine-rich venom proteins altered upon DENV infection of *A. aegypti* mosquitoes (11). Cysteine-rich venom proteins are secretory proteins that are mostly found in the fluids of animal venoms acting on ion channels (24). Londono-Renteria et al. found that among the cysteine-rich venom proteins, only AAEL000379 was enriched in DENV-infected mosquitoes and the rest did not change noticeably. Silencing the gene led to an increase in replication of DENV (23). Alteration of the cysteine-rich venom proteins commonly found in the case of different flaviviruses indicates their possible importance in replication of these viruses. Further studies are required to determine the role that these proteins play in ZIKV-infected mosquitoes specifically.

In another study with DENV-2 and *A. aegypti* in which deep sequencing of carcass, midgut, and salivary glands with one replicate per pooled sample was used, transcript levels of infected and noninfected tissues were compared at 1, 4, and 14 dpi, which showed differential abundance of 397 genes (11). We reanalyzed the raw data from the study using the same pipeline as we used for our study. While comparative analysis of the study with ours cannot properly be made due to differences in the samples (tissues versus whole mosquitoes) and times postinfection, in total, we found 199 genes commonly altered between DENV-2 and ZIKV infections, some with the same directional change in expression (Table S4).

10.1128/mSphere.00456-17.5 TABLE S4  Comparison of transcripts modulated by ZIKV to those modulated by dengue virus identified by Bonizzoni et al. (11). Download TABLE S4, XLSX file, 0.1 MB. Copyright © 2017 Etebari et al. 2017 Etebari et al. This content is distributed under the terms of the Creative Commons Attribution 4.0 International license.

A number of immune-related genes were mostly enriched at 7 dpi in ZIKV-infected mosquitoes. Toll was enriched only at 7 dpi by 2-fold. Twelve leucine-rich immune proteins were mostly enriched at 7 dpi by 4- to 16-fold. Phenol oxidase (AAEL010919), which was not changed upon DENV infection, was depleted by 2-fold at 2 dpi but enriched by 8- to 9-fold at 7 and 14 dpi in ZIKV-infected mosquitoes. Components of the JAK/STAT pathway, such as Dome and Hop, were not induced in ZIKV-infected mosquitoes. Interestingly, induction of the JAK/STAT pathway specifically in the fat body of *A. aegypti* mosquitoes by overexpressing Dome or Hop did not lead to increased resistance to ZIKV infection (25). This result and the lack of induction of the pathway in our study suggest that the JAK/STAT pathway may not be involved in ZIKV-mosquito interaction. Further, major genes involved in the RNA interference (RNAi) pathway, such as Dicer-1, Dicer-2, or any of the Argonaut genes, also did not change upon ZIKV infection in this study.

### Gene ontology.

**FIG 5:**
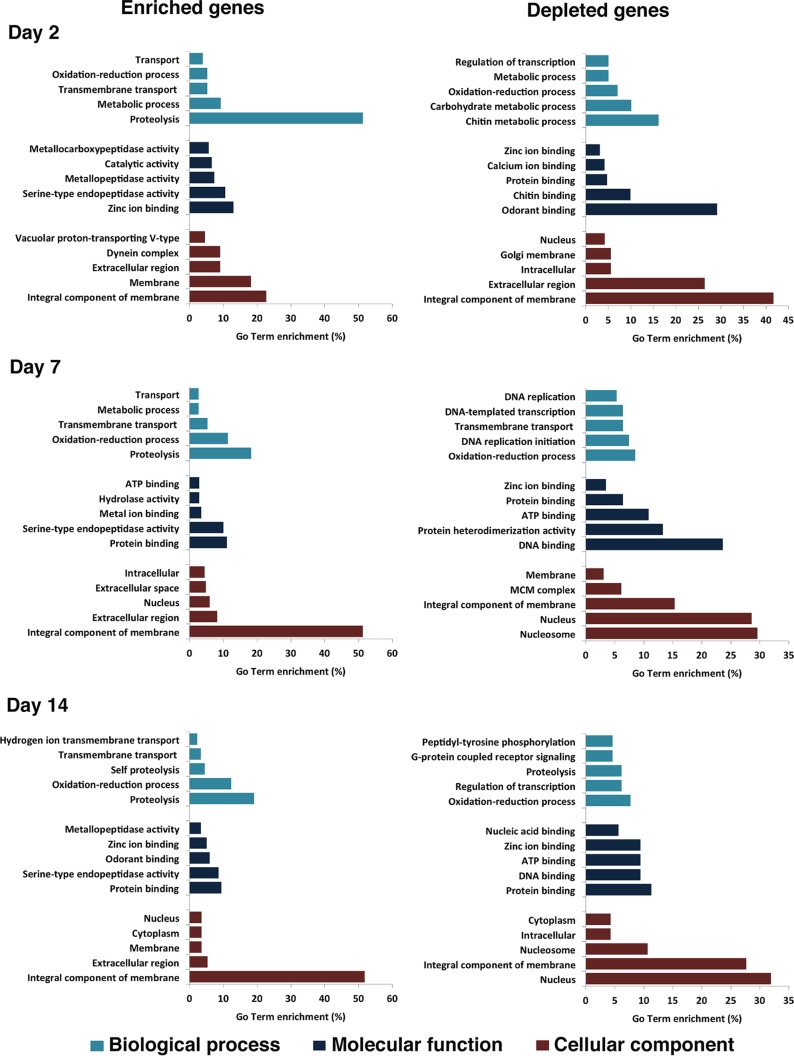
GO term enrichment analysis of differentially expressed genes in response to ZIKV infection in three categories of biological process, molecular function, and cellular component for enriched and depleted genes at 2, 7, and 14 days postinfection.

All the differentially expressed host genes were submitted to Blast2GO for gene ontology (GO) analysis. This analysis identified 126, 68, and 33 GO terms in biological process, molecular function, and cellular components, respectively (Table S5). GO analysis of enriched genes at different times postinfection showed that they were mostly related to proteolysis, zinc ion/protein binding, and integral components of membranes (Fig. 5). Among the depleted genes, the highest categories were more variable, with day 2 having chitin metabolic process, odorant binding, and integral components of membranes; day 7 having oxidation-reduction process, DNA binding, and nucleosome; and day 14 having oxidation-reduction process, protein binding, and nucleus (Fig. 5). In support of our earlier observation (Fig. S1), odorant binding transcripts were depleted at day 2 but enriched at day 14 (Fig. 5). In *A. aegypti*, differentially expressed genes upon infection with DENV, WNV, and YFV belonged to various cellular processes, such as metabolic processes, ion binding, peptidase activity, and transport (13), which are also among the GO terms identified in differentially abundant transcripts in the ZIKV-infected mosquitoes (Fig. 5). The genes commonly altered upon ZIKV and DENV infections that are listed in Table S4 were mostly in proteolysis, oxidation-reduction process, and transmembrane transport from biological process; serine-type endopeptidase activity and protein binding from molecular function; and integral component of membrane, nucleus, and extracellular region from cellular component (Table S4).

10.1128/mSphere.00456-17.6 TABLE S5  Gene ontology analysis for transcripts differentially regulated by ZIKV. Download TABLE S5, XLSX file, 0.1 MB. Copyright © 2017 Etebari et al. 2017 Etebari et al. This content is distributed under the terms of the Creative Commons Attribution 4.0 International license.

### miRNA target genes.

Recently, we identified 17 *A. aegypti* microRNAs (miRNAs) altered upon ZIKV infection at the same time points at which RNA-Seq was conducted (2, 7, and 14 dpi) (16). Comparative analysis of the altered mRNAs and the 17 miRNAs with opposite trends in abundance revealed that 53 of the differentially expressed mRNAs could potentially be regulated by 11 out of the 17 differentially abundant miRNAs (Table S6). However, there is growing evidence that miRNAs could also positively regulate their target genes (26, 27), which are not listed in the table. Further, the analysis showed that some miRNAs have multiple potential target genes as expected (e.g., miR-309a has 19 target genes and miR-981-5p has 12 target genes). Gene ontology analysis of the target genes indicated that the majority of the genes are involved in oxidation-reduction process and integral component of membrane within the biological process and cellular component terms (Table S6).

10.1128/mSphere.00456-17.7 TABLE S6  ZIKV-regulated transcripts potentially affected by miRNAs identified by Saldaña et al. (16). Download TABLE S6, XLSX file, 0.04 MB. Copyright © 2017 Etebari et al. 2017 Etebari et al. This content is distributed under the terms of the Creative Commons Attribution 4.0 International license.

### lincRNAs change upon ZIKV infection.

Long intergenic noncoding RNAs (lincRNAs) are transcripts that are longer than 200 nucleotides (nt) but do not code for any proteins; however, they are transcribed the same way as mRNAs (28), i.e., they have a poly(A) tail and therefore are enriched in transcriptomic data produced following mRNA isolation and sequencing. Similar to small noncoding RNAs, the main function of lincRNAs is regulation of gene expression, involved in various processes such as genomic imprinting and cell differentiation (29), epigenetically and non-epigenetically based gene regulation (30), activation and differentiation of immune cells (31), and, relevantly, virus-host interactions (32–36).

**FIG 6:**
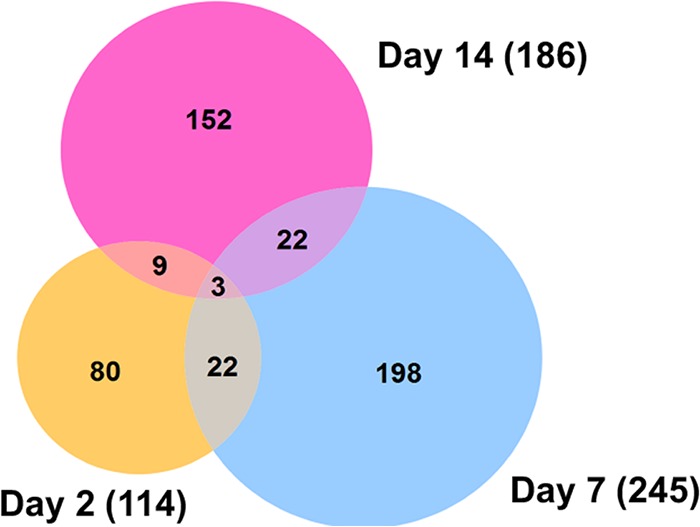
Venn diagram representing the number of differentially expressed lincRNAs at three different time points post-ZIKV infection (fold change of >2 and *P* value of <0.05). The majority of altered lincRNAs were found at 7 dpi, and 56 out of these lincRNAs showed significant alteration at least at two time points.

We recently reported 3,482 putative lincRNAs from *A. aegypti* (32). In this study, we found that, in total, 486 lincRNAs were differentially expressed in response to ZIKV infection in at least one time point postinfection (fold change of >2 and *P* value of <0.05). Similar to mRNAs (Fig. 3), the majority of altered lincRNAs were found at 7 dpi, and 56 out of these lincRNAs showed significant alteration at least in two time points (Fig. 6; Table S7). The Euclidean distance was calculated for each time point based on its lincRNA fold changes. Differentially expressed lincRNAs at 7 dpi (116.83) and 14 dpi (75.30) showed more correlation than, or similar fold change patterns as, those of 2 dpi (180.86). Only lincRNAs 656, 1385, and 3105 were differentially expressed and showed the same fold change pattern among the three time points. In our previous study, we also found that the transcript levels of 421 *A. aegypti* lincRNAs were altered due to DENV-2 infection. Comparison of those with the ones identified in this study showed that about 80 of them were also differentially expressed in ZIKV-infected samples (Table S7), and these could be common lincRNAs involved in flavivirus-mosquito interactions.

10.1128/mSphere.00456-17.8 TABLE S7  Differentially expressed lincRNAs in response to ZIKV at 2, 7, and 14 dpi. Download TABLE S7, XLSX file, 0.1 MB. Copyright © 2017 Etebari et al. 2017 Etebari et al. This content is distributed under the terms of the Creative Commons Attribution 4.0 International license.

### Conclusions.

Overall, our results showed large changes in the transcriptome of *A. aegypti* mosquitoes upon ZIKV infection, in both coding and long noncoding RNAs. The majority of transcriptional changes occurred at 7 dpi, with the genes mostly involved in metabolic process, cellular process, and proteolysis. We found some overlaps of transcriptional alterations in the case of other flavivirus infections in *A. aegypti*, but unlike those, immune genes were not altered to the same extent. In regard to lincRNAs, out of 486 lincRNAs changed in ZIKV-infected mosquitoes, 80 of them overlapped those of DENV-infected mosquitoes, indicating possible conserved functions of the lincRNAs in flavivirus-mosquito interactions. A drawback of this study is that whole mosquitoes were used, which means that changes at the tissue levels could have been overlooked due to the dilution factor of mixing all tissues; however, the outcomes provide a global overview of transcriptional response of *A. aegypti* to ZIKV infection and can be utilized in determining potential proviral and antiviral host factors.

## MATERIALS AND METHODS

### Ethics statement.

ZIKV, which was originally isolated from an *A. aegypti* mosquito (Chiapas State, Mexico), was obtained from the World Reference Center for Emerging Viruses and Arboviruses at the University of Texas Medical Branch (Galveston, TX). Experimental work with the virus was approved by the University of Texas Medical Branch Institutional Biosafety Committee (reference number 2016055).

### Mosquito infections with Zika virus.

We used excess RNA from samples generated recently to investigate miRNA profiles in ZIKV-infected *A. aegypti* mosquitoes (16). Briefly, 4- to 6-day-old female *A. aegypti* mosquitoes (Galveston strain) were orally infected with ZIKV (Mex 1-7 strain) at 2 × 10^5^ focus-forming units (FFU)/ml in a sheep blood meal (Colorado Serum Company). Infected mosquitoes were collected at 2, 7, and 14 days postinfection (dpi), and RNA was extracted from them using the mirVana RNA extraction kit (Life Technologies), applying the protocol for extraction of total RNA. Viral infection in mosquitoes was confirmed by TaqMan quantitative PCR (qPCR) on an ABI StepOnePlus machine (Applied Biosystems) (16). For all time points, three independent pools were used to create libraries for infected and uninfected samples. Uninfected mosquitoes were fed with ZIKV-free blood, collected at the same time points, and processed as described above. The dynamics of infection in mosquitoes was shown in Fig. S1 in the work of Saldaña et al. (16).

### Library preparations and sequencing.

All samples were quantified using a Qubit fluorescence assay (Thermo Scientific). Total RNA quality was assessed using an RNA 6000 chip on an Agilent 2100 Bioanalyzer (Agilent Technologies).

Total RNA (1.0 μg) was poly(A)^+^ selected and fragmented using divalent cations and heat (94°C, 8 min). The NEBNext Ultra II RNA library kit (New England Biolabs) was used for RNA-Seq library construction. Fragmented poly(A)^+^ RNA samples were converted to cDNA by random primed synthesis using ProtoScript II reverse transcriptase (New England Biolabs). After second-strand synthesis, the double-stranded DNAs were treated with T4 DNA polymerase and 5′ phosphorylated, and then an adenine residue was added to the 3′ ends of the DNA. Adapters were then ligated to the ends of these target template DNAs. After ligation, the template DNAs were amplified (5 to 9 cycles) using primers specific to each of the noncomplementary sequences in the adapters. This created a library of DNA templates that have nonhomologous 5′ and 3′ ends. A qPCR analysis was performed to determine the template concentration of each library. Reference standards cloned from a HeLa S3 RNA-Seq library were used in the qPCR analysis. Cluster formation was performed using 15.5 to 17 billion templates per lane using the Illumina cBot v3 system. Sequencing by synthesis, with paired-end 50-base reads, was performed on an Illumina HiSeq 1500 sequencer using a protocol recommended by the manufacturer.

### RNA-Seq data analysis.

The CLC Genomics Workbench version 10.1.1 was used for bioinformatics analyses in this study. RNA-Seq analysis was done by mapping next-generation sequencing reads and distributing and counting the reads across genes and transcripts. The latest assembly of the *A. aegypti* genome (GCF_000004015.4) was used as a reference. All libraries were trimmed from sequencing primers and adapter sequences. Low-quality reads (quality score below 0.05) and reads with more than 2 ambiguous nucleotides were discarded. Clean reads were subjected to an RNA-Seq analysis toolbox for mapping reads to the reference genome with mismatch, insertion, and deletion costs of 2, 3, and 3, respectively. Mapping was performed with stringent criteria and allowed a length fraction of 0.8 in mapping parameter, in which at least 80% of nucleotides in a read must be aligned to the reference genome. The minimum similarity between the aligned region of the read and the reference sequence was set at 80%.

Principal-component analysis (PCA) graphs were produced for each time point after ZIKV infection between control and infected samples to identify any outlying samples for quality control. The expression levels used as input were normalized log count per million (cpm) values.

The relative expression levels were produced as RPKM (*r*eads *p*er *k*ilobase of exon model per *m*illion mapped reads) values, which take into account the relative size of the transcripts and use the mapped-read data sets only to determine relative transcript abundance. To explore genes with differential expression profiles between two samples, CLC Genomic Workbench uses multifactorial statistics based on a negative binomial generalized linear model (GLM). Each gene is modeled by a separate GLM, and this approach allows us to fit curves to expression values without assuming that the error on the values is normally distributed. The TMM (trimmed mean of M values) normalization method was applied on all data sets to calculate effective library sizes, which were then used as part of the per-sample normalization (37).

The Wald test was also used to compare each sample with its control group to test whether a given coefficient is nonzero. We considered genes with more than a 2-fold change and a false discovery rate (FDR) of less than 0.05 as statistically significantly modulated genes.

We previously reported 3,482 putative long intergenic noncoding RNAs (lincRNAs) from *A. aegypti* using a stringent filtering pipeline to remove transcripts that may potentially encode proteins (32). The expression profile of lincRNAs was also generated for each sample similar to the approach described above.

To identify the host transcriptomic response to two different flaviviruses, we compared altered gene profiles in previously published DENV-infected *A. aegypti* libraries (11) with our ZIKV-infected samples. The relevant RNA-Seq data (SRA058076) were downloaded from the NCBI Sequence Read Archive. The libraries were treated in the same way as described above to identify differentially expressed *A. aegypti* gene profiles in response to DENV.

### GO analysis.

All differentially expressed genes were uploaded to the Blast2GO server for functional annotation and GO analysis. We used Blast and InterProScan algorithms to reveal the GO terms of differentially expressed sequences. More abundant terms were computed for each category of molecular function, biological process, and cellular components. Blast2GO has integrated the FatiGO package for statistical assessment, and this package uses Fisher’s exact test.

### Identification of miRNA target genes.

We screened all differentially expressed mRNAs to identify potential miRNA targets among them. If selected mRNAs did not have complete annotations such as clear 5′ untranslated region (UTR), open reading frame (ORF), and 3′ UTR, the region before the ORF start codon (300 bp) and after the stop codon (500 bp) for each mRNA was considered 5′ UTR and 3′ UTR, respectively. We used three different algorithms, including RNA22 (38), miRanda (39), and RNAhybrid (40), to predict potential miRNA binding sites on genes altered by ZIKV. We previously described this approach and parameters for setting each tool, but to increase the level of confidence, we selected those binding sites which were predicted by all three algorithms for further analysis (41).

### RT-qPCR analysis of mRNAs.

qPCR validations were done using the same RNA that was used for RNA-Seq. RNA from ZIKV-positive samples was pooled (*n =* 5) for time points 2, 7, and 14 dpi and treated with amplification-grade DNase I (Invitrogen). Total RNA was reverse transcribed using the amfiRivert cDNA synthesis Platinum master mix (GenDepot, Barker, TX, USA) containing a mixture of oligo(dT)_18_ and random hexamers. Real-time quantification was performed in a StepOnePlus instrument (Applied Biosystems, Foster City, CA) in a 10-µl reaction mixture containing 1:10-diluted cDNA template, 1× PowerUp SYBR green master mix (Applied Biosystems), and 1 µM (each) primer. The analysis was performed using the threshold cycle (ΔΔ*C*_*T*_) (Livak) method (42). Three independent biological replicates were conducted, and all PCRs were performed in duplicate. The ribosomal protein S7 gene (43) was used for normalization of cDNA templates. Primer sequences are listed in Table S8 in the supplemental material.

10.1128/mSphere.00456-17.9 TABLE S8  List of primers used in the study. Download TABLE S8, XLSX file, 0.1 MB. Copyright © 2017 Etebari et al. 2017 Etebari et al. This content is distributed under the terms of the Creative Commons Attribution 4.0 International license.

### Accession number(s).

The accession number for the raw and trimmed sequencing data reported here is GEO GSE102939.

## References

[1] Dick GW, Kitchen SF, Haddow AJ 1952 Zika virus. I. Isolations and serological specificity. Trans R Soc Trop Med Hyg 46:509–520. doi:10.1016/0035-9203(52)90042-4. 12995440

[2] Song BH, Yun SI, Woolley M, Lee YM 2017 Zika virus: history, epidemiology, transmission, and clinical presentation. J Neuroimmunol 308:50–64. doi:10.1016/j.jneuroim.2017.03.001. 28285789

[3] Miner JJ, Diamond MS 2017 Zika virus pathogenesis and tissue tropism. Cell Host Microbe 21:134–142. doi:10.1016/j.chom.2017.01.004. 28182948 PMC5328190

[4] Weaver SC, Costa F, Garcia-Blanco MA, Ko AI, Ribeiro GS, Saade G, Shi PY, Vasilakis N 2016 Zika virus: history, emergence, biology, and prospects for control. Antiviral Res 130:69–80. doi:10.1016/j.antiviral.2016.03.010. 26996139 PMC4851879

[5] Blair CD, Olson KE 2014 Mosquito immune responses to arbovirus infections. Curr Opin Insect Sci 3:22–29. doi:10.1016/j.cois.2014.07.005. 25401084 PMC4228475

[6] Xi Z, Ramirez JL, Dimopoulos G 2008 The *Aedes aegypti* Toll pathway controls dengue virus infection. PLoS Pathog 4:e1000098. doi:10.1371/journal.ppat.1000098. 18604274 PMC2435278

[7] Sánchez-Vargas I, Scott JC, Poole-Smith BK, Franz AW, Barbosa-Solomieu V, Wilusz J, Olson KE, Blair CD 2009 Dengue virus type 2 infections of *Aedes aegypti* are modulated by the mosquito’s RNA interference pathway. PLoS Pathog 5:e1000299. doi:10.1371/journal.ppat.1000299. 19214215 PMC2633610

[8] Sim S, Dimopoulos G 2010 Dengue virus inhibits immune responses in *Aedes aegypti* cells. PLoS One 5:e10678. doi:10.1371/journal.pone.0010678. 20502529 PMC2872661

[9] Tchankouo-Nguetcheu S, Khun H, Pincet L, Roux P, Bahut M, Huerre M, Guette C, Choumet V 2010 Differential protein modulation in midguts of *Aedes aegypti* infected with chikungunya and dengue 2 viruses. PLoS One 5:e13149. doi:10.1371/journal.pone.0013149. 20957153 PMC2950154

[10] Behura SK, Gomez-Machorro C, Harker BW, deBruyn B, Lovin DD, Hemme RR, Mori A, Romero-Severson J, Severson DW 2011 Global cross-talk of genes of the mosquito *Aedes aegypti* in response to dengue virus infection. PLoS Negl Trop Dis 5:e1385. doi:10.1371/journal.pntd.0001385. 22102922 PMC3216916

[11] Bonizzoni M, Dunn WA, Campbell CL, Olson KE, Marinotti O, James AA 2012 Complex modulation of the *Aedes aegypti* transcriptome in response to dengue virus infection. PLoS One 7:e50512. doi:10.1371/journal.pone.0050512. 23209765 PMC3507784

[12] Chauhan C, Behura SK, Debruyn B, Lovin DD, Harker BW, Gomez-Machorro C, Mori A, Romero-Severson J, Severson DW 2012 Comparative expression profiles of midgut genes in dengue virus refractory and susceptible *Aedes aegypti* across critical period for virus infection. PLoS One 7:e47350. doi:10.1371/journal.pone.0047350. 23077596 PMC3471866

[13] Colpitts TM, Cox J, Vanlandingham DL, Feitosa FM, Cheng G, Kurscheid S, Wang P, Krishnan MN, Higgs S, Fikrig E 2011 Alterations in the *Aedes aegypti* transcriptome during infection with West Nile, dengue and yellow fever viruses. PLoS Pathog 7:e1002189. doi:10.1371/journal.ppat.1002189. 21909258 PMC3164632

[14] Campbell CL, Black WC, Hess AM, Foy BD 2008 Comparative genomics of small RNA regulatory pathway components in vector mosquitoes. BMC Genomics 9:425. doi:10.1186/1471-2164-9-425. 18801182 PMC2566310

[15] Etebari K, Osei-Amo S, Blomberg SP, Asgari S 2015 Dengue virus infection alters post-transcriptional modification of microRNAs in the mosquito vector *Aedes aegypti*. Sci Rep 5:15968. doi:10.1038/srep15968. 26514826 PMC4626843

[16] Saldaña MA, Etebari K, Hart CE, Widen SG, Wood TG, Thangamani S, Asgari S, Hughes GL 2017 Zika virus alters the microRNA expression profile and elicits an RNAi response in *Aedes aegypti* mosquitoes. PLoS Negl Trop Dis 11:e0005760. doi:10.1371/journal.pntd.0005760. 28715413 PMC5531668

[17] Maciel-de-Freitas R, Sylvestre G, Gandini M, Koella JC 2013 The influence of dengue virus serotype-2 infection on *Aedes aegypti* (Diptera: Culicidae) motivation and avidity to blood feed. PLoS One 8:e65252. doi:10.1371/journal.pone.0065252. 23755202 PMC3670916

[18] Sylvestre G, Gandini M, Maciel-de-Freitas R 2013 Age-dependent effects of oral infection with dengue virus on *Aedes aegypti* (Diptera: Culicidae) feeding behavior, survival, oviposition success and fecundity. PLoS One 8:e59933. doi:10.1371/journal.pone.0059933. 23555838 PMC3612067

[19] Sim S, Ramirez JL, Dimopoulos G 2012 Dengue virus infection of the *Aedes aegypti* salivary gland and chemosensory apparatus induces genes that modulate infection and blood-feeding behavior. PLoS Pathog 8:e1002631. doi:10.1371/journal.ppat.1002631. 22479185 PMC3315490

[20] Murdock CC, Luckhart S, Cator LJ 2017 Immunity, host physiology, and behaviour in infected vectors. Curr Opin Insect Sci 20:28–33. doi:10.1016/j.cois.2017.03.001. 28602233 PMC5584383

[21] Cator LJ, George J, Blanford S, Murdock CC, Baker TC, Read AF, Thomas MB 2013 ‘Manipulation’ without the parasite: altered feeding behavior of mosquitoes is not dependent on infection with malaria parasites. Proc Biol Sci 280:20130711. doi:10.1098/rspb.2013.0711. PMC3774228 23698008

[22] Cator LJ, Lynch PA, Read AF, Thomas MB 2012 Do malaria parasites manipulate mosquitoes? Trends Parasitol 28:466–470. doi:10.1016/j.pt.2012.08.004. 23044288 PMC3478439

[23] Londono-Renteria B, Troupin A, Conway MJ, Vesely D, Ledizet M, Roundy CM, Cloherty E, Jameson S, Vanlandingham D, Higgs S, Fikrig E, Colpitts TM 2015 Dengue virus infection of *Aedes aegypti* requires a putative cysteine rich venom protein. PLoS Pathog 11:e1005202. doi:10.1371/journal.ppat.1005202. 26491875 PMC4619585

[24] Gibbs GM, O’Bryan MK 2007 Cysteine rich secretory proteins in reproduction and venom. Soc Reprod Fertil Suppl 65:261–267. 17644967

[25] Jupatanakul N, Sim S, Angleró-Rodríguez YI, Souza-Neto J, Das S, Poti KE, Rossi SL, Bergren N, Vasilakis N, Dimopoulos G 2017 Engineered *Aedes aegypti* JAK/STAT pathway-mediated immunity to dengue virus. PLoS Negl Trop Dis 11:e0005187. doi:10.1371/journal.pntd.0005187. 28081143 PMC5230736

[26] Vasudevan S 2012 Posttranscriptional upregulation by microRNAs. RNA 3:311–330. doi:10.1002/wrna.121. 22072587

[27] Vasudevan S, Tong Y, Steitz JA 2007 Switching from repression to activation: microRNAs can up-regulate translation. Science 318:1931–1934. doi:10.1126/science.1149460. 18048652

[28] Clark MB, Mattick JS 2011 Long noncoding RNAs in cell biology. Semin Cell Dev Biol 22:366–376. doi:10.1016/j.semcdb.2011.01.001. 21256239

[29] Bonasio R, Shiekhattar R 2014 Regulation of transcription by long noncoding RNAs. Annu Rev Genet 48:433–455. doi:10.1146/annurev-genet-120213-092323. 25251851 PMC4285387

[30] Mercer TR, Dinger ME, Mattick JS 2009 Long non-coding RNAs: insights into functions. Nat Rev Genet 10:155–159. doi:10.1038/nrg2521. 19188922

[31] Fitzgerald KA, Caffrey DR 2014 Long noncoding RNAs in innate and adaptive immunity. Curr Opin Immunol 26:140–146. doi:10.1016/j.coi.2013.12.001. 24556411 PMC3932021

[32] Etebari K, Asad S, Zhang G, Asgari S 2016 Identification of *Aedes aegypti* long intergenic non-coding RNAs and their association with *Wolbachia* and dengue virus infection. PLoS Negl Trop Dis 10:e0005069. doi:10.1371/journal.pntd.0005069. 27760142 PMC5070814

[33] Lakhotia SC 2012 Long non-coding RNAs coordinate cellular responses to stress. RNA 3:779–796. doi:10.1002/wrna.1135. 22976942

[34] Mizutani R, Wakamatsu A, Tanaka N, Yoshida H, Tochigi N, Suzuki Y, Oonishi T, Tani H, Tano K, Ijiri K, Isogai T, Akimitsu N 2012 Identification and characterization of novel genotoxic stress-inducible nuclear long noncoding RNAs in mammalian cells. PLoS One 7:e34949. doi:10.1371/journal.pone.0034949. 22532836 PMC3330809

[35] Tani H, Onuma Y, Ito Y, Torimura M 2014 Long non-coding RNAs as surrogate indicators for chemical stress responses in human-induced pluripotent stem cells. PLoS One 9:e106282. doi:10.1371/journal.pone.0106282. 25171338 PMC4149554

[36] Winterling C, Koch M, Koeppel M, Garcia-Alcalde F, Karlas A, Meyer TF 2014 Evidence for a crucial role of a host non-coding RNA in influenza A virus replication. RNA Biol 11:66–75. doi:10.4161/rna.27504. 24440876 PMC3929426

[37] Robinson MD, Oshlack A 2010 A scaling normalization method for differential expression analysis of RNA-seq data. Genome Biol 11:R25. doi:10.1186/gb-2010-11-3-r25. 20196867 PMC2864565

[38] Miranda KC, Huynh T, Tay Y, Ang YS, Tam WL, Thomson AM, Lim B, Rigoutsos I 2006 A pattern-based method for the identification of microRNA binding sites and their corresponding heteroduplexes. Cell 126:1203–1217. doi:10.1016/j.cell.2006.07.031. 16990141

[39] Enright AJ, John B, Gaul U, Tuschl T, Sander C, Marks DS 2003 MicroRNA targets in *Drosophila*. Genome Biol 5:R1. doi:10.1186/gb-2003-5-1-r1. 14709173 PMC395733

[40] Krüger J, Rehmsmeier M 2006 RNAhybrid: microRNA target prediction easy, fast and flexible. Nucleic Acids Res 34:W451–W454. doi:10.1093/nar/gkl243. 16845047 PMC1538877

[41] Etebari K, Asgari S 2016 Revised annotation of *Plutella xylostella* microRNAs and their genome-wide target identification. Insect Mol Biol 25:788–799. doi:10.1111/imb.12263. 27515977

[42] Liu Y, Zhou Y, Wu J, Zheng P, Li Y, Zheng X, Puthiyakunnon S, Tu Z, Chen XG 2015 The expression profile of *Aedes albopictus* miRNAs is altered by dengue virus serotype-2 infection. Cell Biosci 5:16. doi:10.1186/s13578-015-0009-y. 25922662 PMC4411651

[43] Isoe J, Collins J, Badgandi H, Day WA, Miesfeld RL 2011 Defects in coatomer protein I (COPI) transport cause blood feeding-induced mortality in yellow fever mosquitoes. Proc Natl Acad Sci U S A 108:E211–E217. doi:10.1073/pnas.1102637108. 21628559 PMC3116422

