# Supplementary material for: Global Transcriptome Analysis of Aedes aegypti Mosquitoes in Response to Zika Virus Infection
Source: mSphere. 2017 Nov 22;2(6):e00456-17. doi: 10.1128/mSphere.00456-17 (PMC5700376; doi:10.1128/mSphere.00456-17)
Supplement: TABLE S1 [file sph006172406st2.docx]

**Table S1.** RNA read summary in ZIKV-infected and non-infected libraries.

| **Name** | **Clean**  **Reads** | **Mapped**  **(Pairs)** | **Mapped**  **(Broken pairs^1^)** | **Not**  **Mapped** | **Counted**  **Fragments^2^** | **Gene** | **Intergenic** |
| --- | --- | --- | --- | --- | --- | --- | --- |
| **Day2-Mock-Rep1** | 49,498,446 | 41,479,002 | 6,420,512 | 1,598,932 | 20,739,501 | 16,565,671 | 4,173,830 |
| **Day2-Mock-Rep2** | 54,611,520 | 46,302,348 | 6,589,249 | 1,719,923 | 23,151,174 | 18,723,003 | 4,428,171 |
| **Day2-Mock-Rep3** | 50,913,398 | 41,076,182 | 8,191,299 | 1,645,917 | 20,538,091 | 16,416,162 | 4,121,929 |
| **Day2-ZIKV-Rep1** | 50,191,342 | 41,293,468 | 7,086,323 | 1,811,551 | 20,646,734 | 16,338,228 | 4,308,506 |
| **Day2-ZIKV-Rep2** | 47,106,824 | 39,305,322 | 6,218,465 | 1,583,037 | 19,652,661 | 15,560,984 | 4,091,677 |
| **Day2-ZIKV-Rep3** | 53,065,836 | 44,476,512 | 6,906,073 | 1,683,251 | 22,238,256 | 17,988,275 | 4,249,981 |
| **Day7-Mock-Rep1** | 54,566,196 | 45,275,168 | 7,603,821 | 1,687,207 | 22,637,584 | 18,676,707 | 3,960,877 |
| **Day7-Mock-Rep2** | 43,486,502 | 36,544,896 | 5,559,495 | 1,382,111 | 18,272,448 | 15,213,846 | 3,058,602 |
| **Day7-Mock-Rep3** | 54,829,994 | 47,613,426 | 5,618,732 | 1,597,836 | 23,806,713 | 19,958,906 | 3,847,807 |
| **Day7-ZIKV-Rep1** | 60,486,566 | 51,809,304 | 6,866,280 | 1,810,982 | 25,904,652 | 21,611,991 | 4,292,661 |
| **Day7-ZIKV-Rep2** | 56,603,564 | 48,043,240 | 6,828,587 | 1,731,737 | 24,021,620 | 19,976,153 | 4,045,467 |
| **Day7-ZIKV-Rep3** | 48,053,642 | 40,332,826 | 6,210,618 | 1,510,198 | 20,166,413 | 16,769,433 | 3,396,980 |
| **Day14-Mock-Rep1** | 49,454,994 | 42,057,568 | 5,903,212 | 1,494,214 | 21,028,784 | 17,686,088 | 3,342,696 |
| **Day14-Mock-Rep2** | 49,654,732 | 41,720,998 | 6,424,692 | 1,509,042 | 20,860,499 | 17,670,812 | 3,189,687 |
| **Day14-Mock-Rep3** | 46,363,650 | 39,151,162 | 5,671,062 | 1,541,426 | 19,575,581 | 16,125,908 | 3,449,673 |
| **Day14-ZIKV-Rep1** | 49,758,224 | 42,184,452 | 5,934,499 | 1,639,273 | 21,092,226 | 17,488,948 | 3,603,278 |
| **Day14-ZIKV-Rep2** | 53,678,614 | 44,809,158 | 6,977,408 | 1,892,048 | 22,404,579 | 18,100,645 | 4,303,934 |
| **Day14-ZIKV-Rep3** | 46,407,592 | 39,562,848 | 5,343,715 | 1,501,029 | 19,781,424 | 16,052,968 | 3,728,456 |

^1^When a pair is broken, either because only one read in the pair matches, or because the distance or relative orientation is wrong.

^2^When two reads map as an intact pair, they are considered as one fragment.
