# Supplementary figures and images for: Global Transcriptome Analysis of Aedes aegypti Mosquitoes in Response to Zika Virus Infection
Source: mSphere. 2017 Nov 22;2(6):e00456-17. doi: 10.1128/mSphere.00456-17 (PMC5700376; doi:10.1128/mSphere.00456-17)

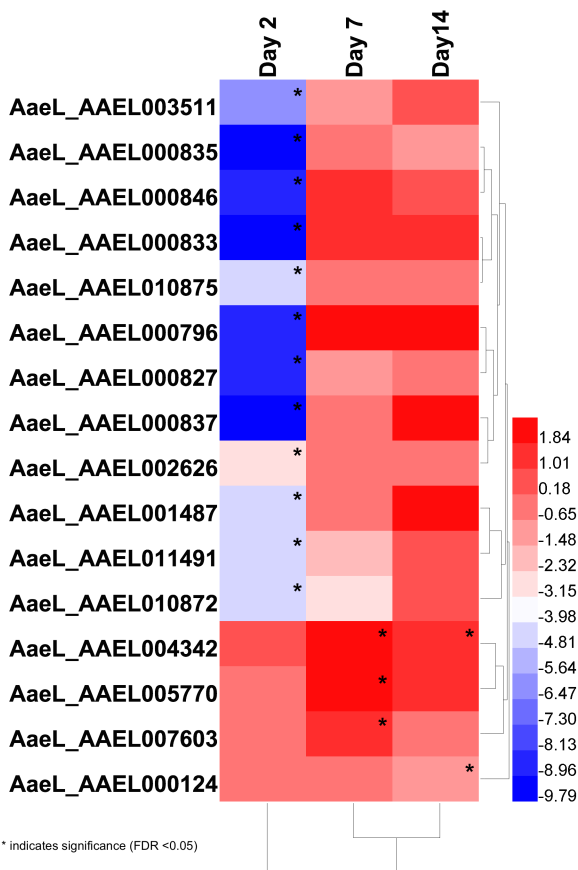

Supplement: FIG S1 [file sph006172406sf1.pdf]
